# Phase I/II dose-finding study of nanoparticle albumin-bound paclitaxel (*nab*®-Paclitaxel) plus Cisplatin as Treatment for Metastatic Nasopharyngeal Carcinoma

**DOI:** 10.1186/s12885-016-2517-5

**Published:** 2016-07-13

**Authors:** Yan Huang, Wenhua Liang, Yunpeng Yang, Liping Zhao, Hongyun Zhao, Xuan Wu, Yuanyuan Zhao, Yang Zhang, Li Zhang

**Affiliations:** Sun Yat-sen University Cancer Center, Yuexiu, Guangzhou, China; State Key Laboratory of Oncology in South China, Guangzhou, China; Collaborative Innovation Center for Cancer Medicine, Guangzhou, China; The First Affiliated Hospital of Guangzhou Medical University, Guangzhou, China; Shaoguan Yuebei People Hospital, Shaoguan, China; Shenzhen Hospital of Peking Univeristy, Shenzhen, China; Department of Medical Oncology, Cancer Center, Sun Yat-Sen University, 651 Dongfeng Road, East, Guangzhou, 510060 People’s Republic of China

## Abstract

**Background:**

This phase I/II study aimed to determine the maximum tolerated dose (MTD) of nanoparticle albumin-bound paclitaxel (*nab*^®^-paclitaxel) plus cisplatin as treatment for metastatic nasopharyngeal carcinoma (NPC).

**Methods:**

Patients were enrolled into 1 of 3 dose cohorts, each with 21-day treatment cycles: 1) intravenous (IV) *nab*-paclitaxel 260 mg/m^2^ on day 1; 2) IV *nab*-paclitaxel 140 mg/m^2^ on days 1 and 8; 3) IV *nab*-paclitaxel 100 mg/m^2^ on days 1, 8, and 15. All patients received IV cisplatin 75 mg/m^2^ on day 1. Treatment continued for 4–6 cycles, or until progression or unacceptable toxicity. If more than one-third of the patients in a cohort experienced a dose-limiting toxicity (DLT), the dose used in the previous cohort would be designated the MTD. Secreted protein acidic and rich in cysteine (SPARC) expression was detected by immunohistochemistry staining.

**Results:**

Sixty-nine patients were enrolled, of whom 64 and 67 were eligible for efficacy and safety analysis, respectively. Two DLTs occurred in cohort 1 (grade 4 febrile neutropenia, grade 3 myalgia), none occurred in cohort 2, and 2 occurred in cohort 3 (both grade 3 fatigue). The MTD was not reached. Partial responses were achieved by 42 patients, 15 had stable disease, and 7 had progressive disease, giving an overall response rate of 66 %. Median progression-free survival was 9 months (95 % CI, 6–12 months). Grade ≥ 3 adverse events were mainly hematologic. There was no significant difference between the 3 cohorts with respect to efficacy or safety. Biomarker analyses indicated that stromal, rather than tumoral, SPARC may predict the response to nab-paclitaxel in NPC.

**Conclusions:**

Our findings suggest that *nab*-paclitaxel plus cisplatin is a highly active regimen with moderate toxicity for the treatment of metastatic NPC, which warrants further investigation in a phase III study.

**Trial registrations:**

ClinicalTrials.gov ID: NCT01735409. The trial was registered on November 20th, 2012.

**Electronic supplementary material:**

The online version of this article (doi:10.1186/s12885-016-2517-5) contains supplementary material, which is available to authorized users.

## Background

Nasopharyngeal carcinoma (NPC) has a particularly high incidence in Southern China (annual incidence > 20/100,000 population [1–3]). Risk factors for NPC include, but are not limited to, Chinese ethnicity, male gender, family history or a genetic predisposition, smoking, and Epstein-Barr virus (EBV) infection [1, 2]. Platinum-based chemotherapy is the backbone of treatment for metastatic or recurrent NPC [4–6], with cisplatin or, less commonly, carboplatin typically being administered with 1 or 2 other active chemotherapeutic agents [4–6].

When combined with platinum-containing chemotherapy, paclitaxel has been shown to be effective in the treatment of advanced or metastatic NPC [7–14]. Indeed, paclitaxel plus cisplatin or carboplatin combinations are now considered standard treatment options [4–6]. Traditional solvent-based paclitaxel is highly hydrophobic, and is therefore formulated in a mixture of polyoxyethylated castor oil (Cremophor EL) and ethanol to facilitate intravenous (IV) infusion [15]. Nanoparticle albumin-bound paclitaxel (*nab*^®^-paclitaxel; ABRAXANE, Celgene, Summit, NJ, USA) is a water-soluble form of paclitaxel linked to albumin nanoparticles [16]. In a number of metastatic solid tumors, *nab*-paclitaxel has shown similar or superior efficacy to traditional solvent-based paclitaxel, but with an improved safety profile [17–20]. Indeed, its approval in combination with carboplatin for the treatment of locally advanced metastatic non-small cell lung cancer was based on clinical trial results demonstrating significantly improved response rates versus solvent-based paclitaxel plus carboplatin [19]. As *nab*-paclitaxel is associated with a lower incidence of neurological toxicities than solvent-based paclitaxel [19], and in view of the potentially increased clinical activity of cisplatin versus carboplatin [21, 22], we decided to evaluate *nab*-paclitaxel in combination with cisplatin in the treatment of metastatic NPC.

This phase I/II, dose-finding study (ClinicalTrials.gov registration ID: NCT01735409) was designed to investigate the safety (especially with regard to neurological effects), tolerability, and antitumor activity of 3 different doses/dosing schedules of *nab*-paclitaxel when combined with cisplatin for the treatment of metastatic NPC. As elevated expression of secreted protein acidic and rich in cysteine (SPARC) is associated with metastasis and a poor prognosis in NPC [23], evaluation of the relationship between SPARC expression and the clinical activity of *nab*-paclitaxel was an exploratory objective of the study. Investigation of the correlation between post-treatment EBV-DNA copy number and clinical outcomes was also an exploratory objective.

## Methods

### Study design

This was a single-center, single-arm, non-randomized, open-label, phase I/II trial, conducted at the Sun Yat-sen University Cancer Center in Guangzhou, China. Eligible patients were enrolled into 3 dose cohorts, each with 21-day treatment cycles: cisplatin 75 mg/m^2^ IV on day 1 with sufficient hydration, plus *nab*-paclitaxel IV (30-min infusion) at doses of: 260 mg/m^2^ on day 1 (cohort 1, dose intensity 260 mg/m^2^ per cycle); 140 mg/m^2^ on days 1 and 8 (cohort 2, dose intensity 280 mg/m^2^ per cycle); or 100 mg/m^2^ on days 1, 8, and 15 (cohort 3, dose intensity 300 mg/m^2^ per cycle). Treatment was administered for 4–6 cycles, or until disease progression or unacceptable toxicity.

It was planned that 23 patients would be enrolled into each cohort. If more than one-third of patients in a given cohort experienced a dose-limiting toxicity (DLT) during all treatment cycles, enrollment would be stopped and the dose used in the previous cohort would be designated as the maximum tolerated dose (MTD). At the initiation stage, we followed the routine mode of dose escalation studies: we enrolled a total of 3 patients into cohort 1. After the 3 patients in this cohort completed the designated cycles of treatment, and no more than 1 DLT occurred, we started cohort 2 and enrolled another 3 patients for dose checking (the same for cohort 3). The cohorts that have completed the initiation stage then entered the expansion stage and additional 20 participants were recruited. Eligible patients were sequentially assigned to all active cohorts (either in initiation stage or expansion stage) one by one. If more than 1 patient experienced DLT in the initiation stage or more than 1/3 of total patients experienced DLT in the expanding stage of any cohort, the corresponding cohort will be closed and the remaining quota of the cohort would be re-assigned to the previous dose cohort.

DLTs were defined as any of the following: National Cancer Institute Common Terminology Criteria for Adverse Events (NCI-CTCAE) grade 4 neutropenia lasting for > 7 days; neutropenia with fever (defined as an absolute neutrophil count [ANC] < 1 × 10^9^/L with a body temperature of ≥ 38.3 °C); grade 4 thrombocytopenia, or grade 3 thrombocytopenia with hemorrhage; grade ≥ 3 neuropathy; or any other grade ≥ 3 non-hematologic toxicity that did not resolve following symptomatic treatment. Concomitant medications without antineoplastic activity were permitted, but their use was recorded.

The study was conducted according to the Declaration of Helsinki, Good Clinical Practice guidelines, and local regulatory requirements. The Ethics Committee of Sun Yat-sen University approved the protocol. All patients provided written informed consent.

Treatment could be delayed for a maximum of 2 weeks to allow recovery from toxicity. For dose adjustments, if patients suffered from grade 4 neutropenia or thrombocytopenia, a 25 % reduction in nab-paclitaxel was applied in the subsequent cycle/administration. Subsequent dose escalation to the original dosage was allowed providing the patient tolerated the doses given at the 75 % level. For non-hematological toxicities, nab-paclitaxel and cisplatin were reduced to a 75 % dose if there were grade 3 toxicities, and the patient went off-study with grade 4 toxicities, excluding those due to nausea/vomiting or alopecia. Patients went off-study if they suffered from grade 3 or worse neuropathy. Subsequent dose escalation was not allowed. A maximum of 2 dose reductions per patient was allowed.

### Patients

Patients aged ≥ 18 years with a histologically proven diagnosis of NPC and evidence of metastatic disease were enrolled. Principal inclusion criteria were: previous failure or intolerance of standard treatment (prior chemotherapy or radiotherapy up to one line) for advanced NPC, or ineligibility for standard therapy (intermediate radiotherapy for local disease); an Eastern Cooperative Oncology Group performance status (ECOG PS) of 0–2; at least 1 measurable evaluable lesion; life expectancy of ≥ 12 weeks; adequate hematologic (ANC > 1.5 × 10^9^/L, platelet count > 100 × 10^9^/L, and hemoglobin ≥ 90 g/L), hepatic (aspartate aminotransferase [AST] and alanine aminotransferase [ALT] < 2.5 × upper limit of normal [ULN], and bilirubin < 1.0 × ULN), and renal (serum creatinine < 1.5 × ULN or estimated creatinine clearance ≥ 60 mL/min [calculated using the Cockcroft-Gault formula]; [24]) function; and willingness to provide a biopsy sample for assessment of SPARC expression.

Exclusion criteria included: treatment with either more than 2 prior lines of anticancer therapy for metastatic disease, or any chemotherapy, radiotherapy, or other anticancer therapies within 3 weeks before enrollment; central nervous system metastases; pre-existing peripheral neuropathy of NCI-CTCAE grade ≥ 2; any active, clinically serious infection requiring or likely to require antibiotics for > 4 weeks; a life-threatening medical condition (e.g., congestive heart failure, symptomatic coronary artery disease, or heart block); a history of myocardial infarction within 3 months before enrollment; prior or current immunodeficiency; a history of allergy to paclitaxel or docetaxel; or a previous or concurrent malignancy other than NPC (except cervical carcinoma in situ, treated basal cell carcinoma, superficial bladder tumors [Ta, Tis, T1], or any cancer curatively treated > 3 years prior to study entry).

### Endpoints

#### Primary endpoint

The primary endpoint was MTD (phase I setting). The primary efficacy endpoint, (phase II setting) which was used to estimate sample size, was the objective response rate (ORR; defined as the proportion of patients with a complete response [CR] or partial response [PR]) according to Response Evaluation Criteria in Solid Tumors (RECIST) version 1.1 [25]. Tumor assessment was undertaken every 2 cycles, with best responses being recorded. Responses were assessed by the investigator only.

#### Secondary endpoints

Secondary endpoints included: disease control rate (DCR; defined as the proportion of patients with a CR, PR, or best response of stable disease [SD]), and progression-free survival (PFS) measured from the date of first infusion until the date of progression or death.

### Assessments

#### Safety assessments

Safety and tolerability were assessed by adverse event (AE) monitoring and evaluation of vital signs, hepatic and renal function, electrocardiograms, blood counts, and changes in electrolytes. AEs were graded according to NCI-CTCAE version 4.0.

#### Efficacy assessments

Antitumor activity was evaluated in the response-evaluable population, defined as all patients whom completed 4–6 cycles of treatment with post-baseline response assessments. Safety endpoints were assessed in the safety population (all patients who received ≥ 1 dose of study medication).

#### Assessment of EBV-DNA copy number

EBV-DNA copy number was evaluated at baseline and after each chemotherapy cycle (before administration of any study medication), using real-time, quantitative, fluorescence-based polymerase chain reaction (PCR). The primer and probe sequences are as follows: AGTCTTCTGTCCTCCAGGCAA (forward), ACAGAGGGCCTGTCCACCG (reverse), FAM-CACTGTCTGTAAAGTCCAGCCTCC-TAMRA (probe).

#### Assessment of SPARC expression

Immunohistochemical assessment of SPARC expression was carried out at the end of enrollment. All the tissue samples were stored samples that were obtained from the primary lesion or metastatic lymph nodes before curative radiotherapy or first-line chemotherapy. SPARC were marked using mouse monoclonal antibody (Life Technologies^®^, clone No., ON1-1). Expression was quantified using H-score [26] for tumoral SPARC and Z-score [27] for stromal SPARC.

### Statistical analyses

#### Sample size estimation

The clinical activity of *nab*-paclitaxel was assessed using historical controls. The response rate of the most commonly used standard regimen of 5-fluorouracil and cisplatin for the treatment of advanced NPC is 60 % [3]. Superiority of *nab*-paclitaxel plus cisplatin versus historical controls would be established if the ORR reached 70 %, based on a value of half the bilateral 95 % CI, 50–90 %. To give a significance level (α) of 0.05 with 80 % statistical power according to the Exact (Clopper-Pearson) approach, it was calculated that the study would need to enroll 69 patients (23 in each cohort).

#### Statistical methods

For comparisons of baseline characteristics or rate of efficacy/toxicity among all cohorts, one-way ANOVA was used to compare the means of continuous variables, non-parametric test was used to compare the median values, and R × C table Chi-square test was used to compare the categorical parameters. For survival analyses, time-to-event distribution was estimated using the Kaplan-Meier method; the *p* value for the stratified log-rank test was obtained from the score test. For all tests, *P* value <0.05 was considered to be significant.

#### Subgroup analyses

Subgroup analyses were performed post-hoc, and included: assessment of ORR in chemotherapy-naïve versus previously treated patients, and in paclitaxel-naïve versus paclitaxel-pretreated patients; comparison of DCR between patients with a > 10 % decrease in EBV-DNA from baseline after cycle 1 versus those with a ≤ 10 % decrease; and analysis of PFS in patients with ≤ 30 %, > 30 to < 50 %, and ≥ 50 % tumor regression (defined as the maximum decrease in the sum of the longest diameters of target lesions), and in patients with complete EBV-DNA clearance at any time during treatment versus those with non-zero trough EBV-DNA. Estimations and comparisons of PFS between the 3 dose cohorts and the exploratory subgroups were carried out by Kaplan-Meier methodology using a log-rank test.

## Results

### Patients

Between January 2013 and January 2014, 69 eligible patients were enrolled: 23 into cohort 1, 22 into cohort 2, and 24 into cohort 3. In total, 64 patients received 4–6 cycles of chemotherapy and were included in the response-evaluable population. Five patients discontinued the study before completing 4–6 cycles of chemotherapy for personal reasons (4 patients after 1 cycle, owing to unwillingness to receive the study therapy, and 1 patient after 2 cycles for economic reasons). No intolerable toxicities were reported in any of the 5 patients who withdrew prematurely. Sixty-seven patients were included in the safety population (2 patients withdrew after receiving only the first dose and were reluctant to provide toxicity report. Thus, we were unable to include them into the safety analysis.).

Demographics and baseline characteristics were balanced between the 3 *nab*-paclitaxel dose cohorts (Table 1).

**Table 1:** Patient demographics and baseline characteristics (safety population)

Characteristic	Cohort 1 (*n* = 23)	Cohort 2 (*n* = 22)	Cohort 3 (*n* = 24)	*P* ^a^
Mean age, years (SD)	47 (10)	50 (10)	45 (12)	0.27
Male, *n* (%)	22 (96)	19 (86)	20 (83)	0.39
WHO histopathological grade, *n* (%)				0.77
Grade I	2 (9)	2 (9)	1 (4)	
Grade II	0	0	0	
Grade III	21 (91)	20 (91)	23 (96)	
Current or past smoker, *n* (%)	13 (57)	15 (68)	14 (58)	0.69
Alcohol use, *n* (%)	19 (83)	18 (82)	19 (79)	0.95
Metastatic sites, *n* (%)				
Lung	8 (35)	5 (22)	7 (29)	0.67
Liver	8 (35)	10 (45)	13 (54)	0.41
Bone	13 (57)	12 (55)	12 (50)	0.90
Distant lymph nodes	7 (30)	7 (32)	4 (17)	0.43
Multiple sites	10 (44)	10 (46)	10 (42)	0.97
Prior chemotherapy, *n* (%)				
Any chemotherapy	16 (70)	15 (68)	20 (83)	0.43
Paclitaxel	5 (21)	4 (27)	9 (45)	0.49
Treatment history, *n* (%)				0.07
Failed 1st-line chemotherapy	15 (65)	10 (46)	12 (50)	
Recurrence after curative treatment	1 (4)	6 (27)	9 (38)	
Treatment-naïve	7 (30)	6 (27)	3 (13)	
Median cycles of prior lines of chemotherapy (range)	5 (1 to 14)	5 (2 to 8)	4 (1 to 10)	0.75
Mean time since last chemotherapy, months (SD)	4 (4)	7 (11)	7 (8)	0.40
Prior radiotherapy, *n* (%)	10 (44)	11 (50)	17 (71)	0.143

Cohort 1, cisplatin 75 mg/m^2^ day 1 + *nab*-paclitaxel 260 mg/m^2^ day 1 Q3W; Cohort 2, cisplatin 75 mg/m^2^ day 1 + *nab*-paclitaxel 140 mg/m^2^ day 1, 8 Q3W; Cohort 3, cisplatin 75 mg/m^2^ day 1 + *nab*-paclitaxel 100 mg/m^2^ day 1, 8, 15 Q3W

^a^ *P* value for the inter-cohort difference

Q3W, every 3 weeks; SD, standard deviation; WHO, World Health Organization

### Dose-limiting toxicity and maximum tolerated dose

DLTs were observed in 2 patients in each of cohort 1 (grade 4 febrile neutropenia [cycle 2; managed using granulocyte-colony stimulating factor] and grade 3 myalgia [cycle 1; managed using a COX-2 inhibitor]) and cohort 3 (both grade 3 fatigue; cycles 1 and 2). All DLTs resolved and the patients received a 25 % dose reduction of *nab*-paclitaxel in all subsequent cycles. DLTs were not reported in cohort 2. The incidence of DLTs did not differ significantly between the 3 cohorts (cohort 1, 9 %; cohort 2, 0 %; cohort 3, 8 %; *P* = 0.37). As less than one-third of the patients in each cohort experienced a DLT, the MTD was not reached.

### Antitumor activity

**Fig. 1:**
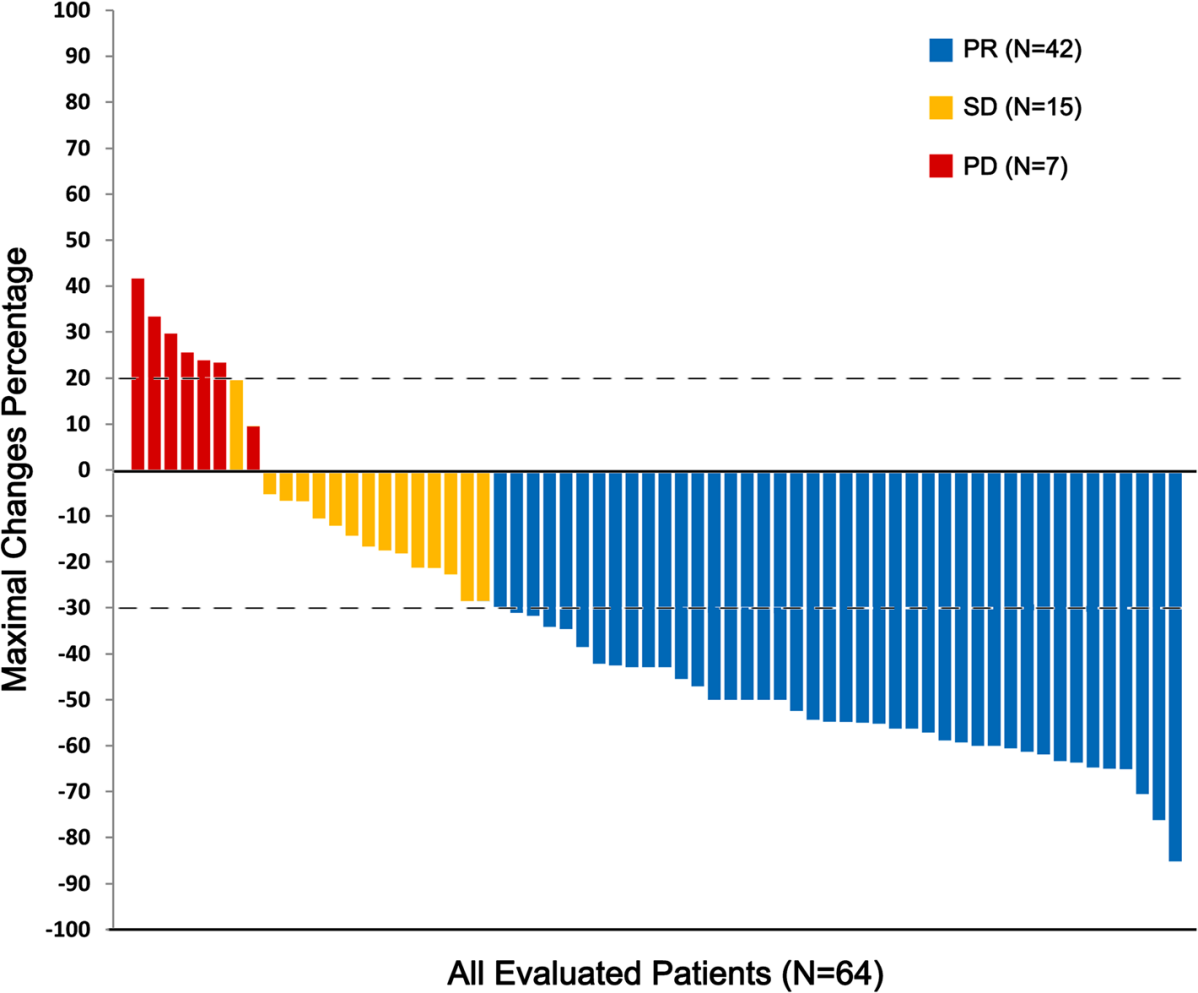
Waterfall plot of maximum percentage change in the sum of the longest diameters of target lesions. PD was defined as an increase of > 20 % in tumor size; PR was defined as a decrease of > 30 % in tumor size

Among the 64 evaluable patients, 42 achieved a PR, 15 had SD, and 7 had progressive disease, resulting in an ORR of 66 % and a DCR of 89 % (Table 2). None of the patients achieved a CR. Fig. 1 shows the maximum change in the sum of the longest diameters of target lesions for all 64 response-evaluable patients. One patient underwent a mixed response, partial response of the pulmonary lesion but progressive disease of the lesion at axillary lymph node, which was considered as overall progressive disease in this study. No statistically significant differences in ORR were observed between the 3 dose cohorts (*P* = 0.94; Table 2).

**Table 2:** Tumor response according to RECIST version 1.1 (response-evaluable population)

Response	Patients, *n* (%)				*P* ^a^
	All patients (*N* = 64)	Cohort 1 (*n* = 22)	Cohort 2 (*n* = 19)	Cohort 3 (*n* = 23)	
CR	0	0	0	0	NA
PR	42 (66)	15 (68)	12 (63)	15 (65)	0.94
SD	15 (23)	5 (23)	4 (21)	6 (26)	0.93
PD	7 (11)	2 (9)	3 (16)	2 (9)	0.72
ORR	42 (66)	15 (68)	12 (63)	15 (65)	0.94
DCR	57 (89)	20 (91)	16 (84)	21 (91)	0.72

Cohort 1, cisplatin 75 mg/m^2^ day 1 + *nab*-paclitaxel 260 mg/m^2^ day 1 Q3W; Cohort 2, cisplatin 75 mg/m^2^ day 1 + *nab*-paclitaxel 140 mg/m^2^ day 1, 8 Q3W; Cohort 3, cisplatin 75 mg/m^2^ day 1 + *nab*-paclitaxel 100 mg/m^2^ day 1, 8, 15 Q3W

^a^ *P* value for the inter-cohort difference

NA, not applicable; PD, progressive disease

In the post-hoc subgroup analyses, chemotherapy-naïve patients achieved a significantly higher ORR than chemotherapy-pretreated patients (100 % [17/17] vs. 53 % [25/47], respectively; *P* < 0.01). The ORR was numerically, but not statistically significantly, higher in paclitaxel-naïve than in paclitaxel-pretreated patients (58 % [18/31] vs. 44 % [7/16], respectively; *P* = 0.35). When stratified according to EBV-DNA copy number, patients with a > 10 % decrease in EBV-DNA from baseline to cycle 1 achieved a DCR of 95 %, whereas, patients with a ≤ 10 % decrease had a DCR of 76 % (*P* = 0.02).

**Fig. 2:**
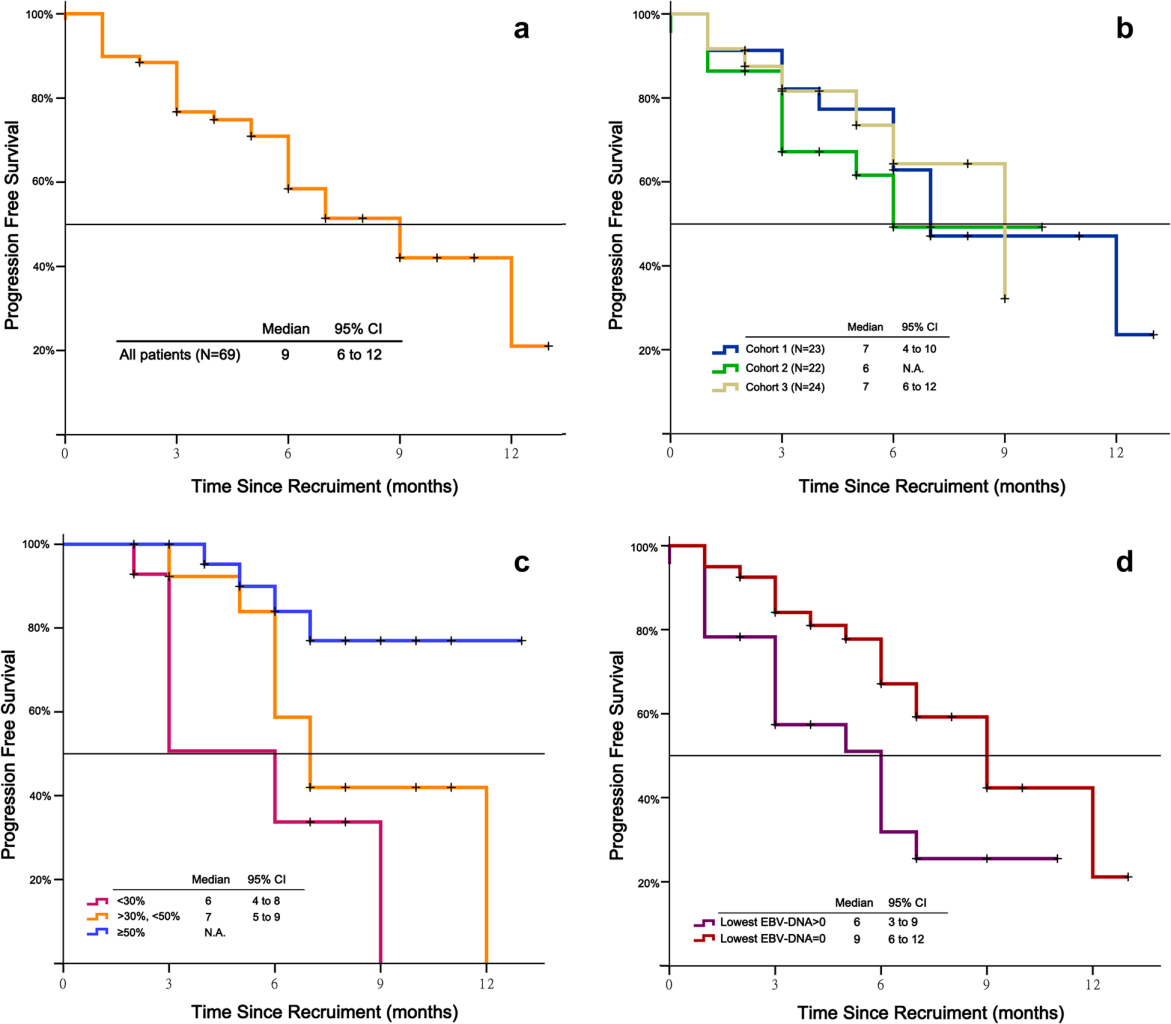
Kaplan-Meier analysis of median progression free survival in: **a**, all study patients; **b**, all 3 *nab*-paclitaxel dose cohorts; **c**, patients with ≤ 30 %, > 30 to < 50 %, and ≥ 50 % maximum decrease in the sum of the longest diameters of target lesions; and **d**, patients with complete EBV-DNA clearance at any time during treatment versus those with non-zero trough EBV-DNA. Cohort 1, cisplatin 75 mg/m^2^ day 1 + *nab*-paclitaxel 260 mg/m^2^ day 1 Q3W; Cohort 2, cisplatin 75 mg/m^2^ day 1 + *nab*-paclitaxel 140 mg/m^2^ day 1, 8 Q3W; Cohort 3, cisplatin 75 mg/m^2^ day 1 + *nab*-paclitaxel 100 mg/m^2^ day 1, 8, 15 Q3W. CI, confidence interval

Median PFS among all evaluable patients was 9 months (95 % CI, 6–12; Fig. 2a), and did not differ significantly between the 3 dose cohorts (7 vs. 6 vs. 9 months, respectively; *P* = 0.91; Fig. 2b). However, median PFS did differ significantly between patients with a ≤ 30 %, > 30 to < 50 %, and ≥ 50 % decrease in the sum of the longest diameters of target lesions (*P* = 0.001; Fig 2c). Additionally, median PFS was significantly longer in patients with complete EBV-DNA clearance at any time during treatment than in those with non-zero trough EBV-DNA (9 vs. 6 months, respectively; *P* = 0.02; Fig. 2d).

**Fig. 3:**
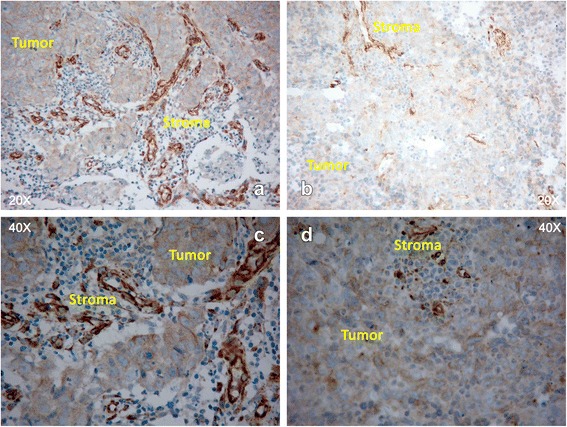
SPARC protein expression. Panels **a** (20× magnification) and **c** (40× magnification) show high stromal expression and low expression on the tumor surface; panels **b** (20× magnification) and **d** (40× magnification) show that stromal expression is scarce, and there is little expression on the tumor surface

Stromal SPARC overexpression predicted significantly better response (74 % [40/54] vs. 29 % [2/7]; odds ratio [OR] 7.1; 95 % CI, 1.2–41.1; *P* = 0.03) and prolonged PFS (9 vs. 3 months; *P* = 0.01). However, SPARC expression on the tumor cell surface alone failed to predict response to treatment (74 % [29/39] vs. 59 % [13/22]; OR 1.4; 95 % CI, 0.7–2.8; *P* = 0.38). Representative examples of tumor samples stained for SPARC expression are shown in Fig. 3.

### Safety

All patients included in the safety analysis experienced at least 1 AE during treatment. AEs of grade ≥ 3 occurred in 51 patients (76 %). No patients discontinued the trial because of AEs. There were a total of 285 events of dose delaying and 21 events of dose reduction in a total of 628 administrations in 316 cycles (note: there are 1, 2 and 3 administrations per cycle in cohort 1, 2 and 3 respectively).

The most commonly reported AEs of any grade were alopecia (97 %) and leucopenia (96 %; Table 3). Grade ≥ 3 AEs were mainly hematologic in nature. The most common were neutropenia (64 %) and leucopenia (54 %; Table 3). Only one case experienced febrile neutropenia but recovered soon after granulocyte colony stimulating factor treatment and empirically prophylactic antibiotic treatment, without any evidence of infection. Non-hematologic grade ≥ 3 AEs that occurred in ≥ 5 % of patients were vomiting (6 %), fatigue (5 %), and skin rash (5 %). No grade 4 non-hematologic events occurred.

**Table 3:** Adverse events (NCI-CTCAE version 4.0) reported during treatment with *nab*-paclitaxel and cisplatin (safety population)

AE	Patients with grade 1–2 AEs, *n* (%)				*P* ^a^	Patients with grade ≥ 3 AEs, *n* (%)				*P* ^a^
	All (*N* = 67)	Cohort 1 (*n* = 23)	Cohort 2 (*n* = 21)	Cohort 3 (*n* = 23)		All (*N* = 67)	Cohort 1 (*n* = 23)	Cohort 2 (*n* = 21)	Cohort 3 (*n* = 23)	
Any type	67 (100)	23 (100)	21 (100)	23 (100)	NA	51 (76)	17 (74)	17 (81)	17 (74)	0.82
Hematologic^b^										
Leucopenia	64 (96)	21 (91)	21 (100)	22 (96)	0.38	36 (54)	10 (43)	13 (62)	13 (57)	0.45
Neutropenia	58 (87)	20 (87)	18 (86)	20 (87)	0.99	43 (64)	15 (65)	14 (67)	14 (61)	0.92
Anemia	55 (82)	16 (70)	19 (90)	20 (87)	0.15	6 (9)	3 (13)	1 (5)	2 (9)	0.63
Thrombocytopenia	18 (27)	8 (35)	5 (24)	5 (22)	0.57	0	0	0	0	NA
Non-hematologic^c^	*N* = 64	*n* = 23	*n* = 18	*n* = 23		*N* = 64	*n* = 23	*n* = 18	*n* = 23	
Alopecia	62 (97)	21 (91)	21 (91)	23 (100)	0.16	0	0	0	0	NA
Fatigue	57 (89)	21 (91)	17 (94)	19 (83)	0.44	3 (5)	1 (4)	0	2 (9)	0.42
Anorexia	53 (83)	21 (91)	16 (89)	16 (70)	0.11	0	0	0	0	NA
Myasthenia	52 (81)	18 (78)	17 (94)	17 (74)	0.22	2 (3)	0	0	2 (9)	0.16
Neuropathy	46 (72)	17 (74)	15 (83)	14 (61)	0.27	0	0	0	0	NA
Constipation	42 (66)	14 (61)	15 (83)	13 (57)	0.17	0	0	0	0	NA
Nausea	40 (63)	15 (65)	13 (72)	12 (52)	0.40	0	0	0	0	NA
Arthralgia	39 (61)	16 (70)	12 (67)	11 (48)	0.27	1 (2)	1 (4)	0	0	0.40
Vomiting	38 (59)	11 (48)	14 (78)	13 (57)	0.14	4 (6)	1 (4)	3 (17)	0	0.08
Skin rash	35 (55)	14 (61)	8 (44)	13 (57)	0.56	3 (5)	2 (9)	1 (6)	0	0.37
Myalgia	34 (53)	10 (43)	10 (56)	14 (61)	0.48	1 (2)	1 (4)	0	0	0.40
Pruritus	32 (50)	15 (65)	5 (28)	12 (52)	0.06	0	0	0	0	NA
Diarrhea	21 (33)	8 (35)	6 (33)	7 (30)	0.95	1 (2)	0	1 (6)	0	0.27
Edema	20 (31)	5 (22)	2 (11)	13 (57)	0.004	0	0	0	0	NA

Cohort 1, cisplatin 75 mg/m^2^ day 1 + *nab*-paclitaxel 260 mg/m^2^ day 1 Q3W; Cohort 2, cisplatin 75 mg/m^2^ day 1 + *nab*-paclitaxel 140 mg/m^2^ day 1, 8 Q3W; Cohort 3, cisplatin 75 mg/m^2^ day 1 + *nab*-paclitaxel 100 mg/m^2^ day 1, 8, 15 Q3W

^a^ *P* value for the inter-cohort difference

^b^Blood samples for hematologic AE assessment were available for 67 patients

^c^Non-hematologic AEs were assessed in the 64 patients who completed 4–6 cycles

There were no significant differences between the 3 cohorts in the incidence of individual AEs (any grade or grade ≥ 3), except for grade 1–2 edema, which was significantly more common in cohort 3 (*P* = 0.004). One patient from cohort 2 died during treatment (between cycles 4 and 5) following a cerebral infarction; this was thought to be partially related to treatment.

## Discussion

Our results demonstrate moderate toxicity and substantial antitumor activity of *nab*-paclitaxel plus cisplatin in metastatic NPC, particularly among chemotherapy-naïve patients.

Among evaluable patients, the ORR was 66 % (100 % in chemotherapy-naïve patients), the DCR was 89 %, and median PFS was 9 months. The high antitumor activity of the *nab*-paclitaxel/cisplatin regimen reflects the known chemosensitivity of NPC [3, 4]. The ORR of 66 % is consistent with response rates reported for standard-of-care, first- (50–90 %) and second-line (22–75 %) platinum-based doublets in metastatic NPC [3]. The 100 % ORR in chemotherapy-naïve patients suggests that *nab*-paclitaxel/cisplatin may be particularly active in frontline use. However, as responses were seen in both paclitaxel-naïve and paclitaxel-pretreated patients, prior treatment with taxane-based therapy should not be considered a barrier to treatment with *nab*-paclitaxel. The median PFS of 9 months is also consistent with data reported for active platinum-based doublets in metastatic or recurrent NPC (median, 4–11 months; [3]). As would be expected, patients in our study with the largest reductions in tumor size had the longest PFS (Fig. 2c).

Toxicities associated with *nab*-paclitaxel and cisplatin were predictable, generally acceptable, and manageable. Only 4 DLTs were reported, and the MTD was not reached. Toxicities associated with *nab*-paclitaxel and cisplatin were mainly hematologic in nature, and were typical of platinum/paclitaxel doublets in metastatic/recurrent NPC and other head and neck carcinomas [7–9, 28–33]. Grade ≥ 3 hematologic toxicities were observed in the majority of patients; however, grade ≥ 3 non-hematologic toxicities were relatively uncommon (incidence ≤ 6 % for individual AEs), and there were no reports of severe neuropathy. Although half the enrolled population had received cisplatin-based first-line therapy, we observed no intolerable aggravation of neuropathy among these patients. The overall incidence of all-grade or grade 3/4 toxicities might be numerically higher in this study than previous reports on nab-paclitaxel regimens [17–20]. There are several potential reasons; first, more than half of all included patients were in 2nd line chemotherapy in this study, patients in this group may be more susceptible to toxicity especially hematological types. Second, all included patients were Chinese. There is a relatively large body of evidence showing that Asian populations experience higher incidence of or more severe toxicities at the standard dose compared with Caucasians [34]. Third, since this is a phase I/II study, we used a self-report form and more intense inquiry strategy to track the toxicity event in order not to miss any of the events we concerned, which might contribute to higher recorded toxicity than some historical data.

To our knowledge, this is the first dose-finding clinical trial to evaluate *nab*-paclitaxel combined with cisplatin alone in metastatic NPC. One important implication of our results is that *nab*-paclitaxel combined with cisplatin is a safe, tolerable regimen, especially with respect to neuropathy. These findings can be projected to the treatment of other malignant tumors, such as non-small cell lung cancer and ovarian cancer.

Previous studies in other indications have shown that *nab*-paclitaxel has an AE profile similar in nature to that of conventional paclitaxel, but with a lower incidence of some AEs (e.g., grade ≥ 3 neutropenia, arthralgia, and myalgia), and without side effects usually attributed to Cremophor EL, such as bronchospasms, hypotension, and hypersensitivity reactions [17–19, 35]. Consistently, no such AEs were observed in our study.

Neither toxicity (except for any-grade edema, which was more common in the highest dose cohort) nor antitumor activity differed significantly between the 3 *nab*-paclitaxel dose cohorts. Based on these findings, and bearing in mind the inconvenience of IV drug infusion, we recommend that the *nab*-paclitaxel dose of 260 mg/m^2^ given on day 1 every 3 weeks should be taken forward into further studies of the *nab*-paclitaxel/cisplatin combination in metastatic NPC.

EBV-DNA levels during treatment have been shown to predict disease progression/relapse and overall survival (OS) in both non-metastatic and metastatic NPC [36–42]. In our study, a reduction in EBV-DNA copy number during treatment with *nab*-paclitaxel and cisplatin predicted both disease control and prolonged PFS. Patients with a > 10 % decrease in EBV-DNA from baseline after cycle 1 had a statistically higher DCR than patients with a ≤ 10 % decrease (95 % vs. 76 %, respectively; *P* = 0.02). Similarly, patients with complete EBV-DNA clearance during treatment had a longer PFS than those with non-zero trough EBV-DNA (median, 9 vs. 6 months, respectively; *P* = 0.02). Overall, these results suggest that changes in EBV-DNA copy number may be a practical surveillance tool in patients with metastatic NPC receiving *nab*-paclitaxel/cisplatin combination treatment.

SPARC overexpression has been associated with a reduced disease-free interval and poorer OS in patients with head and neck cancer [43]. However, due to the interaction between SPARC and albumin-bound drugs, SPARC overexpression may be predictive of improved outcomes following treatment with *nab*-paclitaxel in head and neck cancer and advanced pancreatic cancer [44, 45], although this correlation was not found in NSCLC cell lines [46]. In our study, stromal SPARC overexpression strongly predicted a better response to *nab*-paclitaxel (OR 7.1; 95 % CI, 1.2–41.1; *P* = 0.03) and improved PFS (9 vs. 3 months; *P* = 0.01) than expression at normal levels. By contrast, SPARC expression on the surface of tumor cells was not predictive of a response. This is similar to findings in an earlier phase I/II trial in advanced pancreatic cancer, where only stromal SPARC expression appeared to be an effective marker of increased activity of *nab*-paclitaxel [45]. However, in the phase III Metastatic Pancreatic Adenocarcinoma Clinical Trial (MPACT) of *nab*-paclitaxel plus gemcitabine versus gemcitabine alone, no association was found between stromal, tumor epithelial, or plasma SPARC expression and either survival or ORR in patients with metastatic pancreatic cancer [47]. The contradictory findings in these studies underline that further research is needed to clarify whether, and in which cancer types, SPARC expression may be a valuable tool to predict response to *nab*-paclitaxel.

## Conclusions

In conclusion, the results of the present phase I/II trial suggest that, in patients with metastatic NPC, *nab*-paclitaxel plus cisplatin is a highly active regimen with moderate toxicity. Future studies may provide further evidence to support a role for *nab*-paclitaxel plus cisplatin in this indication. The raw data are available in the (Additional file 1: Table S1).

## Abbreviations

AE, adverse event; ALT, alanine aminotransferase; AST, aspartate aminotransferase; CR, complete response; DCR, disease control rate; DLT, dose-limiting toxicity; ECOG PS, Eastern Cooperative Oncology Group performance status; EBV, Epstein-Barr virus; IV, Intravenous; MTD, maximum tolerated dose; *nab*^®^-paclitaxel, nanoparticle albumin-bound paclitaxel; NPC, nasopharyngeal carcinoma; NCI-CTCAE, National Cancer Institute Common Terminology Criteria for Adverse Events; ORR, objective response rate; OS, overall survival; PR, partial response; PCR, polymerase chain reaction; PFS, progression-free survival; RECIST, Response Evaluation Criteria in Solid Tumors; SPARC, Secreted protein acidic and rich in cysteine; SD, stable disease; ULN, upper limit of normal.

## Additional file

Additional file 1: Raw data that supports the conclusion. (XLS 763 kb)
